# Clinical significance of SOX9 in human non-small cell lung cancer progression and overall patient survival

**DOI:** 10.1186/1756-9966-31-18

**Published:** 2012-03-03

**Authors:** Chun-Hui Zhou, Li-Ping Ye, Shi-Xing Ye, Yan Li, Xin-Yin Zhang, Xin-Yu Xu, Li-Yun Gong

**Affiliations:** 1 Department of Biochemistry and Molecular Biology, School of medicine, ShenZhen University, Shen Zhen, China; 2 Department of Pathology, Second Affiliated Hospital of Guangzhou Medical University, Guangzhou, China

**Keywords:** Non-small cell lung cancer, Prognosis, Biomarker, SOX9

## Abstract

**Background:**

Sex determining region Y (SRY)-related high mobility groupbox 9 (SOX9) is an important transcription factor required for development, which regulates the expression of target genes in the associated pathway. The aim of this study was to describe the expression of SOX9 in human non-small cell lung cancer (NSCLC) and to investigate the association between SOX9 expression and progression of NSCLC.

**Methods:**

SOX9 protein and mRNA expression in normal human pneumonocytes, lung cancer cell lines, and eight pairs of matched lung cancer tissues and their adjacent normal lung tissues were detected by Western blotting and real-time reverse transcription-polymerase chain reaction (RT-PCR). Immunohistochemistry was used to determine SOX9 protein expression in 142 cases of histologically characterized NSCLC. Statistical analyses were applied to test for prognostic and diagnostic associations.

**Results:**

SOX9 in lung cancer cell lines was upregulated at both mRNA and protein levels, and SOX9 mRNA and protein were also elevated in NSCLC tissues compared with levels in corresponding adjacent non-cancerous lung tissues. Immunohistochemical analysis demonstrated a high expression of SOX9 in 74/142 (52.1%) paraffin-embedded archival lung cancer biopsies. Statistical analysis indicated that upregulation of SOX9 was significantly correlated with the histological stage of NSCLC (*P *= 0.017) and that patients with a high SOX9 level exhibited a shorter survival time (*P *< 0.001). Multivariate analysis illustrated that SOX9 upregulation might be an independent prognostic indicator for the survival of patients with NSCLC.

**Conclusions:**

This work shows that SOX9 may serve as a novel and prognostic marker for NSCLC, and play a role during the development and progression of the disease.

## Background

Globally, lung cancer was the most commonly diagnosed cancer and the leading cause of cancer death in males, comprising 13% (1.6 million) of the total cases of cancer and 18% (1.4 million) of total cancer deaths in 2008 [1]. The main clinical types of lung cancer are small cell lung cancer(SCLC) and non-small cell lung cancer (NSCLC). NSCLC represents almost 80% of lung cancer, which is the leading cause of cancer-related death in the world. The most common types of NSCLC are squamous cell lung carcinoma, adenocarcinoma, and large cell lung cancer. Surgical resection with adjuvant chemotherapy is the preferred approach for early stage NSCLC, while patients with advanced NSCLC are usually treated with chemotherapy or radiation therapy. Despite advances in treatment, the prognosis is generally poor. Following complete surgical resection of stage IA disease, 5-year survival of patients is 67%, but the 5-year survival rate of individuals with stage IV NSCLC is below 1% [2]. One reason for such a low survival rate is that patients do not receive treatment early enough in disease progression for it to be effective, which is associated with the high metastasis character of NSCLC. Progression from low- to high -stage lung cancer is related to various molecular alterations. However, the cytogenetic and molecular data on various forms of NSCLC are still being investigated for better understanding the disease. The molecular mechanism underlying the progression of NSCLC requires further research, with a view to basing therapy on molecular signatures within tumors. There is significant clinical value in early detection and provision of effective interventions to treat NSCLC.

Sex determining region Y (SRY)-related high mobility group (HMG)-box 9 (SOX9) shares 70% amino acid homology to SRY through its HMG box, the domains of which are involved in the regulation of DNA-dependent processes, such as transcription and replication [3]. SOX9 function was first identified as a key regulator of cartilage and male gonad development, with mutations in *SOX9 *causing campomelic dysplasia and autosomal sex reversal [4,5]. Subsequently, it emerged that SOX9 has been found to be upregulated in several tumor types, such as lung adenocarcinoma, breast carcinoma, colorectal cancer, and prostate cancer [6-9]. However, the clinical and functional significance of SOX9 expression has not been characterized previously in all stages of NSCLC despite the recently reported correlation between upregulation of SOX9 and lung adenocarcinoma, and its association with cancer cell growth [6]. In the present study, SOX9 expression was characterized in all stages of NSCLC from early to advanced. This study found that the expression level of SOX9 was correlated strongly with the histological stage and the survival time of NSCLC patients. In addition, the usefulness of SOX9 as a prognostic factor was evaluated by multivariate analysis. The data revealed that SOX9 could be a lung cancer-associated molecule with a prognostic value.

## Methods

### Cell lines

Primary normal lung epithelial cells (NLEC) were established according to a previously report [10]. In brief, surgical specimens from normal lung were promptly removed and transported aseptically in Hanks' solution (Invitrogen, Carlsbad, CA) with 100 units/ml penicillin, and 100 μg/ml streptomycin (Invitrogen, Carlsbad, CA) and 5 μg/ml gentamicin (Invitrogen, Carlsbad, CA). The tissue specimens were incubated with 1.5 units/ml dispase (Roche Molecular Biochemicals) at 4°C overnight, and the epithelium was dissected away and incubated with trypsin (Invitrogen, Carlsbad, CA). The reaction was stopped with soybean trypsin inhibitor (Sigma, Saint Louis, MI) and centrifuged. The pellet was resuspended in keratinocyte-SFM medium (KSFM) (Invitrogen, Carlsbad, CA) supplemented with 40 μg/ml bovine pituitary extract (Invitrogen, Carlsbad, CA), 1.0 ng/ml EGF (Invitrogen, Carlsbad, CA), 100 units/ml penicillin, 100 μg/ml streptomycin (Invitrogen, Carlsbad, CA), 5 μg/ml gentamycin, and 100 units/ml nyastatin (Invitrogen, Carlsbad, CA). NEEC cells were grown at 37°C and 5% CO2 with KSFM, with 40 μg/ml bovine pituitary extract, 1.0 ng/ml EGF, 100 units/ml penicillin, and 100 μg/ml streptomycin. Lung cancer cell lines, including SK-MES-1, NCI-H460, NCI-H358, NCI-H1650, NCI-H1975, NCI-H596 and PAa, were provided by American Type Culture Collection (ATCC) and grown in the Dulbecco's Modified Eagle Medium (DMEM) (Invitrogen, Carlsbad, USA) with 10% fetal bovine serum (Invitrogen) at 37°C in a 5% CO_2 _atmosphere.

### Patients and tissue specimens

This study was conducted on a total of 142 paraffin-embedded lung cancer specimens, which were diagnosed histopathologically at Second Affiliated Hospital of Guangzhou Medical College from 2006 to 2010. Prior patient consent and approval from the Institutional Research Ethics Committee were obtained to use these clinical materials for research purposes. Clinical information on these samples is described in Table 1. Percentage tumor purity in sections adjacent to the regions used for RNA extraction was estimated during routine histopathological analysis. Normal lung tissues were obtained from First Affiliated Hospital of Shenzhen University by collecting donations from individuals who died in traffic accidents and were confirmed to be free of any prior pathologically detectable conditions. The tumors were staged according to the 7^th ^edition of the Cancer Stage Manual written by the American Joint Committee on Cancer (AJCC) [11].

**Table 1:** Clinicopathologic characteristics of studied patient and expression of SOX9 in NSCLC

	No. (%)
**Gender**	
Male	103(72.5)
Female	39(27.5)
**Age (years)**	
≤ 65	89(62.7)
>65	53(37.3)
Pathology	
Squamous cell carcinoma	47(33.1)
Adenocarcinoma	68(47.9)
Adenosquamous carcinoma	27(19.0)
**NSCLC histology (AJCC grade)**	
I	32(22.5)
II	25(17.6)
III	58(40.8)
IV	27(19.0)
**Survival (n = 89)**	
Alive	33(37.1)
Dead	56(62.9)
**Survial time of low expression**	
Mean 31.70	
Median 28.50	
**Survival time of high expression**	
Mean 24.84	
Median 24.00	
**Expression of SOX9**	
Negative	7(4.9)
Positive	135(95.1)
Low expression	68(47.9)
High expression	74(52.1)

### RNA extraction and real-time reverse transcription-polymerase chain reaction

Total RNA from cultured cells was extracted using the TRIzol reagent (Invitrogen) and purified using the purelink RNA Mini Kit (Invitrogen) according to the manufacturer's instructions. Real-time reverse transcription-polymerase chain reaction (RT-PCR) was employed to quantify the change of SOX9 mRNA level in lung cancer cell lines compared with that in normal human pneumonocytes. Real-time RT-PCR primers and probes for SOX9 and glyceraldehyde-3-phosphate dehydrogenase (GAPDH) were designed with the assistance of the Primer Express version 2.0 software (Applied Biosystems).

#### Primer sequences

SOX9 forward primer: 5'-CGAAATCAACGAGAAACTGGAC-3', SOX9 reverse primer: 5'-ATTTAGCACACTGATCACACG-3', SOX9 probe 5'-(FAM) CCATCATCCTCCACGCTTGCTCTG (TAMRA)-3', GAPDH forward primer: 5'-GACTCATGACCACAGTCCATGC-3', GAPDH reverse primer: 5'-AGAGGCAGGGATGATGTTCTG-3', GAPDH probe 5'-(FAM) CATCACTGCCACCCAGAAGACTGTG (TAMRA)-3'.

Expression data were normalized to the housekeeping gene GAPDH and calculated as 2-^[(Ct of *gene*) - (Ct of *GAPDH*)]^, where Ct represents the threshold cycle for each transcript.

### Western blotting

Cells were harvested in sampling buffer and boiled for 10 min. The procedure was perfomed similarly to previously described methods [12]. Protein concentration was determined with the bicinchoninic acid (BCA) assay (Pierce, Rockford, USA) according to the manufacturer's instructions. Equal amounts of protein were separated electrophoretically on 10% sodium dodecyl sulfate (SDS)-polyacrylamide gels and transferred onto polyvinylidene difluoride membranes (Millipore, Bedford, USA). The membrane was probed with an anti-SOX9 rabbit antibody (1:2,000 dilution; Millipore) and incubated with goat anti-rabbit immunoglobulin G (1:50,000 dilution; Pierce). Expression of SOX9 was determined with SuperSignal West Pico Chemiluminescent Substrate (Thermo, USA) according to the manufacturer's suggested protocol. The membranes were stripped and reprobed with an anti-actin mouse monoclonal antibody (1:2,000 dilution; Millipore) as a loading control.

### Immunohistochemistry (IHC)

Immunohistochemical analysis was performed to study altered protein expression in 142 human lung cancer tissues. The procedures were carried out in a similar manner to previously described methods [13]. Paraffin-embedded specimens were cut into 4 μm sections and baked at 65°C for 30 minutes. The sections were deparaffinized with xylenes and rehydrated. Sections were submerged into ethylenediaminetetraacetic acid antigenic retrieval buffer and microwaved for antigenic retrieval. The sections were treated with 3% hydrogen peroxide in methanol to quench the endogenous peroxidase activity, followed by incubation in 1% bovine serum albumin to block non-specific binding. Rabbit anti-SOX9 (1:50 dilution; Millipore) was incubated with the sections at 4°C overnight. Primary antibody was replaced by normal goat serum in the negative controls. After washing, the tissue sections were treated with biotinylated anti-rabbit secondary antibody (Zymed, San Francisco, USA) followed by a further incubation with streptavidin-horseradish peroxidase complex (Zymed). The tissue sections were immersed in 3-amino-9-ethyl carbazole and counterstained using 10% Mayer's hematoxylin, dehydrated, and mounted in Crystal Mount (Sigma). The degree of immunostaining of formalin-fixed, paraffin-embedded sections was viewed and scored separately by two independent investigators, who were blinded to the histopathological features and patient details of the samples. Scores were determined by combining the proportion of positively stained tumor cells and the intensity of staining. The scores given by the two independent investigators were averaged for further comparative evaluation of SOX9 expression. The proportion of positively stained tumor cells was staged as follows: 0 (no positive tumor cells), 1 (<10% positive tumor cells), 2 (10-50% positive tumor cells), and 3 (>50% positive tumor cells). The cells at each intensity of staining were recorded on a scale of 0 (no staining), 1 (weak staining, light yellow), 2 (moderate staining, yellowish brown), and 3 (strong staining, brown). The staining index was calculated as follows: staining index = staining intensity × proportion of positively stained tumor cells. Using this method of assessment, the expression of SOX9 in lung cancers was evaluated using the staining index (scored as 0, 1, 2, 3, 4, 6, or 9). High and low expression of SOX9 were defined using cutoff values based on a measure of heterogeneity with the log-rank test statistics with respect to overall survival; an optimal cutoff value was identified. A staining index score of ≥ 6 was used to define tumors with high expression and a staining index ≤ 4 was used to define tumors with low expression of SOX9.

Immunohistochemical staining for protein expression in tumor and normal tissues was quantitatively analyzed with the Olympus BX51 image analysis system assisted with the CellSens Dimension 1.5 Imaging software. The stained sections were evaluated at × 200 magnification and 10 representative staining fields per section were analyzed to verify the mean absorbance, which represents the strength of staining signals as measured per positive pixels. The mean absorbance data were analyzed statistically using t test to compare the average mean absorbance difference between different groups of tissues; a *P *< 0.05 was considered significant.

### Statistical analysis

All statistical analyses were carried out using the statistical software package, SPSS, version 17.0 (IBM SPSS, Chicago, USA). The χ^2 ^test was used to analyze the relationship between SOX9 expression and the clinicopathological characteristics. Bivariate correlations between study variables were calculated by Spearman rank correlation coefficients. Survival curves were plotted with the Kaplan-Meier method and compared by the log-rank test. Survival data were evaluated using univariate and multivariate Cox regression analyses. In all cases, *P *< 0.05 was considered statistically significant.

## Results

### Increased expression of SOX9 in NSCLC

**Figure 1:**
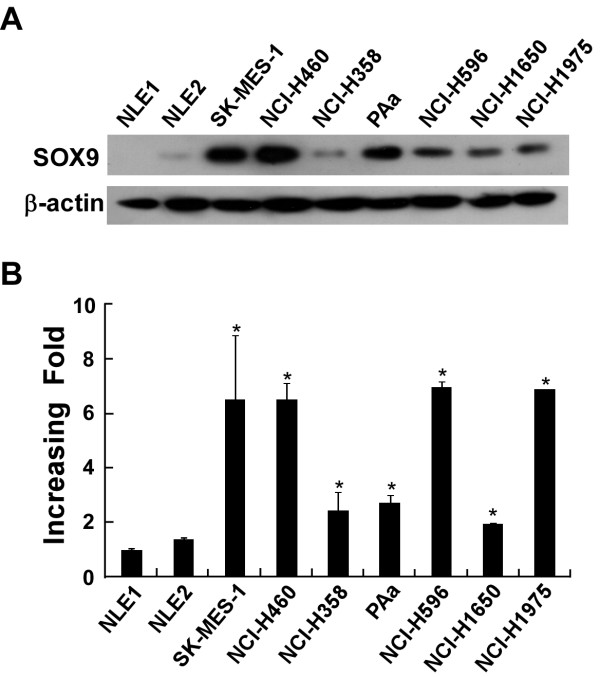
**Expression of SOX9 was elevated in NSCLC cell lines. A and B**. Expression analysis of SOX9 protein and mRNA in normal human pneumonocyte (NLE) and NSCLC cell lines (SK-MES-1, NCI-H460, NCI-H358, PAa, NCI-H596, NCI-H1650, NCI-H1975) by Western blotting **(A) **and real-time RT-PCR **(B)**. Protein expression levels were normalized with β-actin mRNA expression levels were normalized for GAPDH. Bars, SD from three independent experiments.

Western blotting and real-time PCR analyses were performed to determine the levels of SOX9 protein and mRNA, respectively, in primary normal lung epithelial cells (NLEC) and seven NSCLC cell lines: SK-MES-1, NCI-H460, NCI-H358, NCI-H1650, NCI-H1975, NCI-H596, and PAa. All tumor cell lines showed significantly higher levels of SOX9 protein (Figure 1A) and SOX9 mRNA expression (Figure 1B) compared with NLEC, which showed no or marginal SOX9 expression.

**Figure 2:**
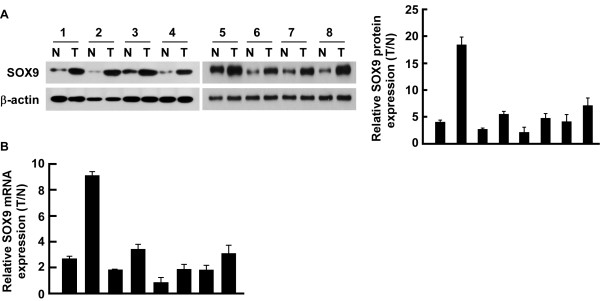
**Differential expression of SOX9 in human NSCLC tissues (T) and their paired normal lung tissue (N), each pair obtained from the same patient**. **A**, Western blotting analysis of SOX9 protein expression in eight paired tumor and normal tissue samples, average tumor/normal ratios of SOX9 protein expression quantified using the LabWorks software. Expression levels were normalized with β-actin. **B**, average tumor/normal ratios of SOX9 expression were quantified by real-time RT-PCR. Expression levels were normalized for GAPDH. Bars, SD from three independent experiments

To determine whether the level of SOX9 is associated with the progression of NSCLC, comparative analysis of SOX9 expression was conducted on eight pairs of matched lung cancer tissue and the non-cancerous tissue adjacent to the malignant lesion using Western blotting and real-time RT-PCR analyses. As shown in Figure 2A, the expression of SOX9 protein was upregulated in all eight human primary NSCLC samples compared with their paired adjacent non-cancerous tissue. Similarly, the mRNA expression level of SOX9 was also upregulated in NSCLC malignant lesions compared with that in the paired adjacent lung tissue (Figure 2B). It should be noted that the level of SOX9 protein in the NSCLC cell lines and clinical lung cancer tissues was paralleled with the mRNA expression level, indicating the possibility that the increase of SOX9 in NSCLC might be largely attributable to regulation at the mRNA level.

### Correlation between increased expression of SOX9 and malignancy of NSCLC

**Figure 3:**
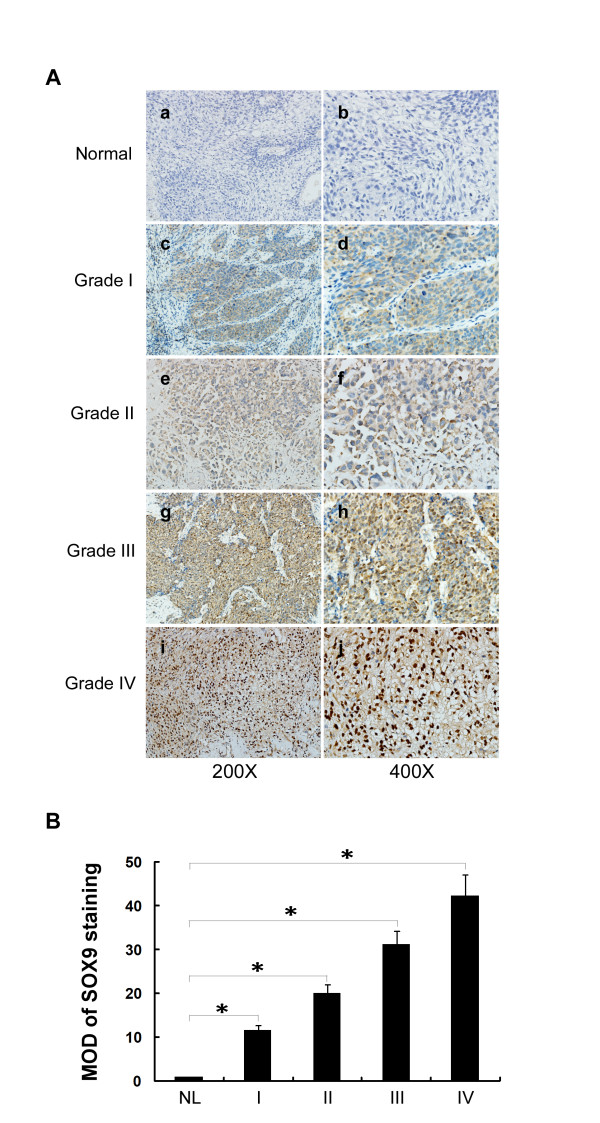
**Immunohistochemical analysis of SOX9 expression in normal lung tissue and primary NSCLC**. **A**, thin sections of paraffin-embedded specimens of a total of 2 normal lung tissues and 142 primary NSCLC specimens including AJCC grade I to IV NSCLC samples were stained by immunohistochemistry using an anti-SOX9 antibody. a and b, normal lung tissue; c and d, AJCC grade I NSCLC; e and f, AJCC grade II NSCLC; g and h, AJCC grade III NSCLC; i and j, AJCC grade IV. Amplification, 200 (a, c, e, g, and i) and 400 (b, d, f, h and j). Immunohistochemical analyses were done two independent times on each of the samples with similar results. **B**, statistical quantification of the average mean absorbance of SOX9 staining between normal lung tissues (4 cases) and NSCLC specimens of different AJCC grades (30 random cases per grade). Average mean absorbance of SOX9 staining increases as NSCLC progresses to higher grades. *, *P *< 0.01.

To determine whether the expression level of SOX9 protein is associated with the histological characteristics of NSCLC, 142 paraffin-embedded, archived NSCLC clinical specimens, which included 32 cases of stage I, 25 cases of stage II, 58 cases of stage III, and 27 cases of stage IV lung cancers, were examined by immunohistochemical staining with an antibody against human SOX9. As shown in Figure 3A, SOX9 was found to be upregulated in NSCLC (c and d, stage I NSCLC; e and f, stage II NSCLC; g and h, stage III NSCLC; and i and j, stage IV NSCLC) compared with that in the normal lung tissue (Figure 3). As summarized in Table 1, SOX9 protein was detected in 135 of 142 (95.1%) cases. High levels of SOX9 were present in areas containing tumor cells in primary NSCLC tissues (Figure 3A, c-j). In contrast, SOX9 was barely detectable in normal lung tissue (Figure 3A and 3B). Quantitative analysis indicated that the average mean absorbance of SOX9 staining in stage I-IV tumors was statistically significantly higher than in normal lung tissue (*P *< 0.01; Figure 3B).

### Expression of SOX9 protein and histological staging of NSCLC

Immunostaining examination of tumor sections obtained from 142 patients showed that positive SOX9 expression was found to be correlated strongly with the clinicopathological stages of the patients' cancer (*P *= 0.022), but no significant relationship was found between age (*P *= 0.382) or gender (*P *= 0.240), or pathology (*P *= 0.312) (Table 2). Spearman correlation analysis revealed a correlation coefficient of 0.200 (*P *= 0.017; Table 3) between SOX9 expression level and the histological grading of NSCLC. Taken together, these observations support the notion that the progression of NSCLC is associated with increased SOX9 expression.

**Table 2:** Correlation between the clinicalpathologic features and expressions of SOX9

Characteristics	*SOX9*		*P*-value
		
	Low or none	High	
Gender			0.382
Male Female	47 21	56 18	
Age (years)			0.240
≤ 65 >65	46 22	43 31	
Pathology			
Squamous cell carcinoma	26	21	0.312
Adenocarcinoma	32	36	
Adenosquamous carcinoma	10	17	
NSCLC histology (AJCC grade)			0.022
I and II III and IV	34 34	23 51	
Survival (n = 89)			0.040
Alive Dead	21 23	12 33	

**Table 3:** Spearman correlation analysis between SOX9 and clinical pathologic factors

Variables	SOX9	
	
	Spearman Correlation	*P*-Value
Gender	-0.083	0.325
Age	0.098	0.247
NSCLC histology (AJCC grade)	0.200	0.017
Survival	-0.239	0.024

### Association between SOX9 expression and patient prognosis

**Figure 4:**
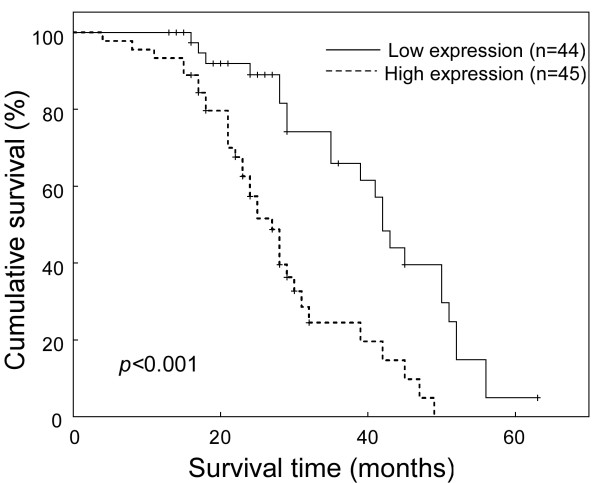
**Kaplan-Meier curves with univariate analyses (log-rank) for patients with low SOX9-expressing (bold line) versus high SOX9-expressing tumors (dotted line)**. The cumulative 3-year survival rate was 65.9% in the low SOX9 expression group (n = 44), whereas it was only 24.5% in the high SOX9 expression group (n = 45).

The statistical analysis presented in Table 2 revealed an inverse correlation between SOX9 level and patient survival (*P *= 0.040). Spearman analysis also showed a correlation coefficient of -0.239 (*P *= 0.024; Table 3) between SOX9 and patient survival. Log-rank test and Kaplan-Meier analysis were also applied to evaluate further the effect of SOX9 expression and histological staging of lung cancer on survival. The log-rank test showed that the expression level of SOX9 protein in NSCLC was correlated significantly with patients' survival time (*P *< 0.001), with a correlation coefficient of -0.262 (Figure 4; Table 4). As shown in Figure 4, the cumulative 3-year survival rate was 65.9% in the low-SOX9 expression group (n = 44), and 24.5% in the high-SOX9 expression group (n = 45). The multivariate survival analysis shown in Table 4 indicated that SOX9 expression level was an independent prognostic factor in the assessment of patient outcomes.

**Table 4:** Univariate and multivariate analysis of different prognostic parameters in patients with NSCLC by Cox-regression analysis

	Univariate analysis			Multivariate analysis		
	
	No. patients	*P*	Regression coefficient (SE)	*P*	Relative risk	95% confidence interval
Age		0.147	0.020(0.014)	0.779	1.004	0.976-1.032
≤ 65	53					
>65	36					
NSCLC histology (AJCC grade)						
I	33	0.016	0.354(0.146)	0.049	1.368	1.001-1.868
II						
III	56					
IV						
SOX9		0.000	0.776(0.199)	0.001	2.004	1.350-2.974
Low	44					
High	45					

The expression level of SOX9 protein in NSCLC was significantly correlated with patients' survival time (*P *< 0.05); the correlation coefficient was -0.262, indicating that higher levels of SOX9 expression was correlated with shorter survival time.

**Figure 5:**
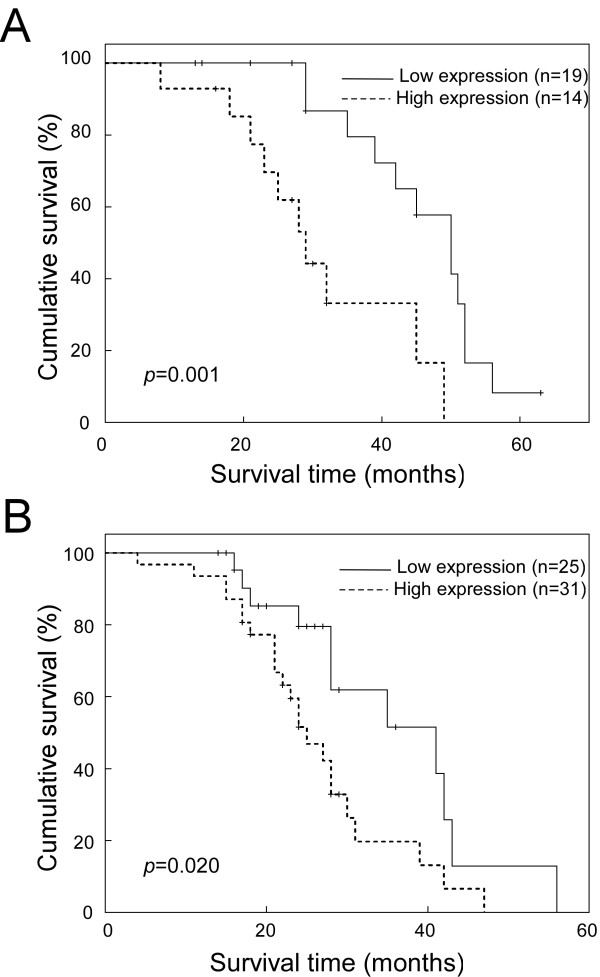
**Kaplan-Meier analysis showing the overall survival of NSCLC patients categorized according to the AJCC grades and status of SOX9 expression**. The statistical significance of the difference between curves of SOX9 high-expressing and low-expressing patients was compared within subgroups of AJCC grades I+II (**A**) and III+IV (**B**). *P *values were calculated by the log-rank test.

The prognostic value of SOX9 expression in different subgroups of NSCLC patients was stratified in relation to the histological staging. A significant correlation was found between high SOX9 expression and shorter overall survival time in AJCC-graded subgroups of NSCLC. Patients with tumors exhibiting high SOX9 expression had significantly shorter overall survival than those with low expression of SOX9 in either stages I + II subgroup (n = 43; *P *= 0.001, log-rank; Figure 5A) or stages III + IV subgroup (n = 56; *P *= 0.020, log-rank; Figure 5B), indicating that SOX9 could be a valuable prognostic marker for NSCLC patients at all disease stages.

## Discussion

The major finding of our study is that the progression of human NSCLC is related to upregulation of SOX9 expression. Although, a previous report has described a correlation between the expression of SOX9 mRNA and protein levels with lung adenocarcinoma [6], this study represents the first demonstration that SOX9 mRNA and protein are upregulated in all stages of human NSCLC and that this degree of upregulation increases as NSCLC progresses to advanced stages.

Recent cogent evidence has provided a link between SOX9 and cancer development and progression [14,15], and the upregulation of SOX9 has been observed in several types of solid tumors, including lung adenocarcinoma, breast carcinoma, colorectal cancer, and prostate cancer [6-9]. In addition, there is marked inhibition of differentiation, coupled with an expanded domain of expression of SOX9 protein in Nmyc overexpressing lung [16]. It has been reported that the induction of SOX9 expression could be induced through various mechanisms. Dysregulation of tissue development pathways can be conducive to cancer initiation and progression. As part of a developmental pathway, elevation of SOX9 in prostate neoplasia promotes tumor cell proliferation [17]. Moreover, as a transcription factor, SOX9 is linked to the hedgehog pathways and may play a role in the development of malignant peripheral nerve sheath tumor in patients [18]. Ling et al. reported that despite SOX9 levels being high during periods of prenatal urothelial development in mouse bladders, SOX9 was diminished and quiescent with maturation after birth, but was rapidly induced by a variety of injuries and urothelial cancer [19]. All these findings suggest that SOX9 may play important roles in cancer development and progression, which prompted the authors to ask whether it is also clinically associated with the progression of NSCLC. To address this question, studies were performed to characterize the expression of SOX9 in NSCLC cell lines and clinical lung cancer tissues. The data show that upregulation of SOX9 mRNA and protein is a common and frequent event in both NSCLC cell lines and human lung cancer tissues. Comparative analyses of SOX9 mRNA and protein in lung cancer tissues and their paired adjacent normal tissue have provided strong support for the identified upregulation of SOX9 in NSCLC. Moderate to strong cytoplasmic staining of SOX9 was displayed in tumor cells from 135/142 (95.1%) paraffin-embedded archived NSCLC biopsy samples in comparison with the adjacent non-cancerous cells, which expressed little, if any, SOX9.

Further analysis of the relationship between SOX9 staining and the clinicopathological characteristics of patients showed a significant correlation between SOX9 expression and the histopathological staging of NSCLC. This revealed that SOX9 levels were higher in advanced stages of the disease, supporting the hypotheses that SOX9 may play a role in the progression of NSCLC and that it could represent a biomarker that identifies subsets of lung-cancer patients with more aggressive disease. It is of particular note that patients with high SOX9 expression had shorter survival time, suggesting the possibility of using SOX9 as a predictor for patient prognosis and survival.

In a more detailed survival study, univariate and multivariate analyses demonstrated that high expression of SOX9 is a predictor of poor prognosis for lung-cancer patients. It is of note that there is a significant correlation between shorter overall survival times of patients and high SOX9 expression in both the early histological stage subgroup (stages I and II) and the late histological stage subgroup (stages III and IV), suggesting that SOX9 may be a useful prognostic marker for all stages of NSCLC.

## Conclusions

Although several lines of evidence have suggested that SOX9 might be involved in cancer development and progression, only a few studies have linked SOX9 to lung cancer. Knockdown of SOX9 has been found to decrease the proliferation rate of lung cancer cell lines and significantly attenuate the tumorigenicity of lung adenocarcinoma [6]. Despite the above finding, the precise pathway that SOX9 uses to inhibit the differentiation of NSCLC and promote lung cancer development and progression remains unclear. Based on the findings from this and other studies, further investigation is warranted to validate whether SOX9 can be used as a novel therapeutic target for NSCLC.

## Competing interests

The authors declare that they have no competing interests.

## Authors' contributions

Chun-Hui Zhou and Li-Ping Ye participated in the data collection, performed the statistical analysis and drafted the manuscript. Shi-Xing Ye assisted with the data collection, Yan-Li, Xin-Yin Zhang, Xin-Yu Xu made substantial contributions to the analysis and interpretation of data, Dr. Li-Yun Gong conceived of the study, participated in its design and coordination and helped to draft the manuscript. All authors read and approved the final manuscript.
